# In-vitro cytotoxicity assessment of carbon-nanodot-conjugated Fe-aminoclay (CD-FeAC) and its bio-imaging applications

**DOI:** 10.1186/s12951-015-0151-z

**Published:** 2015-11-26

**Authors:** Kyoung Suk Kang, Hyun Uk Lee, Moon Il Kim, So Young Park, Sung-Jin Chang, Ji-Ho Park, Yun Suk Huh, Jouhahn Lee, Mino Yang, Young-Chul Lee, Hyun Gyu Park

**Affiliations:** Department of Chemical and Biomolecular Engineering (BK21+ Program), KAIST, 291 Daehakno, Yuseong-gu, Daejeon, 305-701 Republic of Korea; Advanced Nano-Surface Research Group, Korea Basic Science Institute (KBSI), Daejeon, 305-333 Republic of Korea; Department of BioNano Technology, Gachon University, 1342 Seongnamdaero, Sujeong-gu, Seongnam-si, Gyeonggi-do 461-701 Republic of Korea; Department of Chemistry, Chung-Ang University, 84 Heukseok-ro, Dongjak-gu, Seoul, 156-756 Republic of Korea; Department of Bio and Brain Engineering (BK21+ Program), KAIST, 291 Daehakno, Yuseong-gu, Daejeon, 305-701 Republic of Korea; Department of Biological Engineering, College of Engineering, Inha University, Incheon, 402-751 Republic of Korea; Division of Analytical Research, Korea Basic Science Institute (KBSI), Gangneung, 200-701 Republic of Korea

**Keywords:** In-vitro cytotoxicity, Fe-aminoclay (FeAC), Carbon nanodots (CD), Cytotoxicity, Conjugation, Bio-imaging

## Abstract

We have investigated the cytotoxic assay of Fe-aminoclay (FeAC) nanoparticles (NPs) and simultaneous imaging in HeLa cells by photoluminescent carbon nanodots (CD) conjugation. Non-cytotoxic, photostable, and CD NPs are conjugated with cationic FeAC NPs where CD NPs play a role in bio-imaging and FeAC NPs act as a substrate for CD conjugation and help to uptake of NPs into cancer cells due to positively charged surface of FeAC NPs in physiological media. As increase of CD-FeAC NPs loading in HeLa cell in vitro, it showed slight cytotoxicity at 1000 μg/mL but no cytotoxicity for normal cells up to concentration of 1000 μg/mL confirmed by two 3-(4,5-dimethylthiazol-2-yl)-2,5-diphenyltetrazolium bromide (MTT) and neutral red (NR) assays, with further observations by 4′,6-diamidino-2-phenylindole (DAPI) stained confocal microscopy images, possessing that CD-FeAC NPs can be used as potential drug delivery platforms in cancer cells with simultaneous imaging.

**Electronic supplementary material:**

The online version of this article (doi:10.1186/s12951-015-0151-z) contains supplementary material, which is available to authorized users.

**Graphical abstract:**
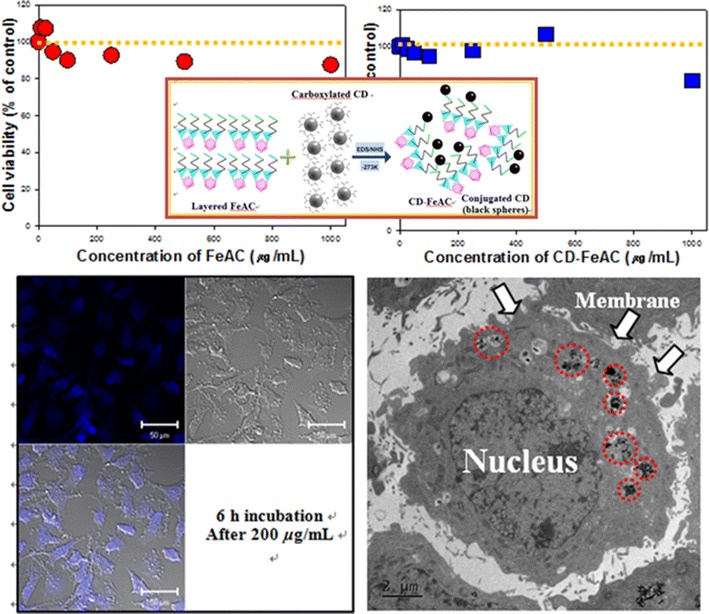
CD conjugation with organo-building blocks of delaminated FeAC NPs

## Background

Over and beyond the various organic [1–6] and inorganic [7–10] nanoparticles (NPs), many hybrid organic–inorganic [11–16] NPs have been intensively researched in biomedical, environmental, and energy applications for their size, shape, charge, and surface chemistry including diverse functionalities. Specific organic-functional groups in many hybrid NPs offer usually unique properties in terms of the accessability and bioactivity of targeting cells or biomolecules in bionanotechnology without some post-functionalization in nanotechnology [17–20].

One candidate of organic–inorganic NPs with covalent-bonded primary amines, namely 3-aminopropyl-functionalized magnesium phyllosiliate [i.e., Mg-aminoclay, formulated as [H_2_N(CH_2_)_3_]_8_Si_8_Mg_6_O_12_(OH)_4_] was developed by one-pot sol–gel reaction under ambient conditions by Mann et al. [21, 22], showing unique interactions of organic-pendents with cell or other molecules [23, 24] in biomedical fields, as well as with heavy metals [25] in environmental applications. This aminoclay structure is composed of tetrahedral brucite (MgO) in the middle, sandwiched by octahedral silica (SiO_2_) as the unit structure in the vertical direction (i.e., 2:1 trioctahedral clay) and a repeated tetrahedral/octahedral structure in pairs, known as the 1:1 dioctahedral structure. Diverse high-density primary amines [–(CH_2_)_3_NH_2_] in octahedral structures have been coined *aminoclays* [26], according to the cationic metals used in their preparation [27, 28].

Recently, organo-building blocks of Mg- and Ca-aminoclays were tested for possible use as drug-delivery carriers, and were found to result in neither cytotoxicity nor inflammation [29]. Further, protonated clusters of Mg-aminoclay with positively charged zeta potential in the wide pH range of 2.0–12.0 [30] were tested as biodistribution and elimination pathways in in vivo mice after Cy 5.0 conjugation with organo-building blocks in delaminated Mg-aminoclay. The results showed fast elimination or excretion of Mg-aminoclay in mice after oral or intravenous injection, respectively, without toxicity [31]. With the exceptions of transparent Mg- and Ca-aminoclays in aqueous solutions, other colored aminoclays have not been tested for cytotoxicity to determine the feasibility of their use in biomedical applications. Importantly, the lack or weak fluorescent-emission intensity of aminoclays has driven research to explore fluorescent imaging for promising drug-delivery-carrier and simultaneous bio-imaging applications in diagnostics and therapeutics.

Carbon nanodots (CD) in zero-dimensional (0D) carbon materials [32–34] in the form of biocompatible and non-toxic fluorescent NPs with properties distinct from those of one-dimensional (1D) carbon nanotubes (CNTs) [12, 35, 36] and two-dimensional (2D) graphene [37–39], are especially intriguing for their photostability and bio-imaging, and contrast-agent applicabilities, Also, CD has a potential of their mass production from organic molecules using eco-friendly preparation methods [34].

## Results

### CD conjugated FeAC (CD-FeAC) NPs

**Fig. 1:**
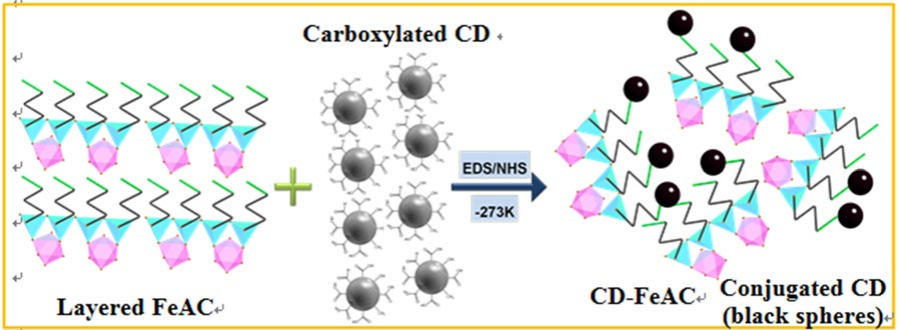
Schematic representation of CD conjugation with organo-building blocks of delaminated FeAC NPs in PBS buffer (0.01 M and pH 7.2) by amide linkage

In the present study, characterizations of water-solubilized FeAC NPs [27, 40–42] and photoluminescent (PL) CD-conjugated FeAC (CD-FeAC) NPs was performed to determine their cytotoxicities in designed cell lines (Table 1). Particularly, the uptake of CD-FeAC NPs in HeLa cells, which is two different-sized and multifunctional NP platforms, was bio-imaged. Schematically (Fig. 1), mesolamellar-stacked FeAC NPs were delaminated in aqueous solution by repulsion of protonated-amine-enriched organo-building blocks of FeAC sheets. CD NPs, contrastingly, were dispersed in aqueous solution by 10 min bath sonication. 1-ethyl-3-(3-dimethylaminopropyl)-carbodiimide/*N*-hydroxysulfosuccinimide (EDS/NHS) conjugation at low temperature afforded organic pendent groups of organo-building blocks of FeAC (i.e., CD-FeAC) NPs without reforming mesolamellar layers.

**Table 1:** Designed system of cell lines used in this study

Cell name	Organism	Tissue	Cell type (morphology)	Disease
HeLa	*Homo sapiens*, human	Cervix	Epithelial (Epithelial)	Adenocarcinoma
A549	*Homo sapiens*, human	Lung	– (Epithelial)	Carcinoma
WI-38	*Homo sapiens*, human	Lung	Fibroblast (Fibroblast)	Normal
WM-266-4	*Homo sapiens*, human	Derived from metastatic site: skin	Melanoma (Epithelial)	Malanoma
CCD-986SK	*Homo sapiens*, human	Skin	Fibroblast (Fibroblast)	Normal
RAG	*Mus musculus*, mouse	Kidney	Amoeboid	Adenocarcinoma

### Rudimental characteristics of FeAC, CD, and CD-FeAC NPs

**Fig. 2:**
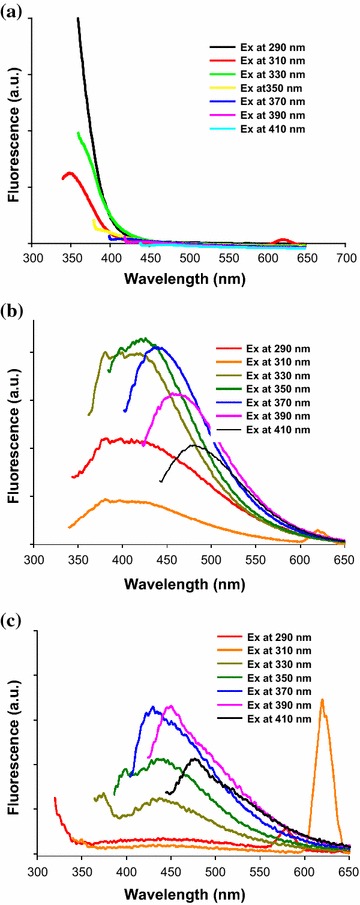
Fluorescent emission spectra of **a** FeAC, **b** CD, and **c** CD-FeAC NPs according to excitation wavelength ranging from 290 to 410 nm with 20 nm wavelength intervals

**Fig. 3:**
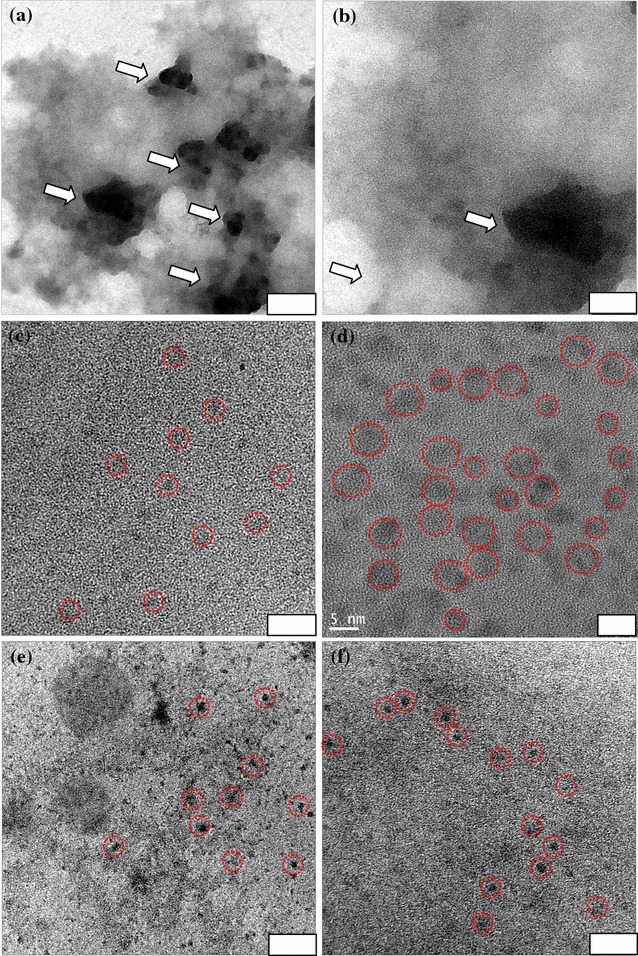
Transmission electron microscopy (TEM) images of **a**, **b** FeAC, **c**, **d** CD, and **e**, **f** CD-FeAC NPs dispersed in PBS buffer at 2.5 mg/mL. Note that in **a**, **b**, the *arrows* indicate the edges of the organo-building blocks of FeAC NPs, and that in **c**–**f**, the *red dotted circles* stand for CD NPs. *Scale bar* **a** 100 nm, **b** 10 nm, **c** 50 nm, **d** 5 nm, **e** 100 nm, **f** 10 nm

The PL spectra of FeAC, CD, and CD-FeAC NPs at 290–410 nm excitation wavelengths were recorded with emission spectra (Fig. 2). The PL intensity of FeAC NPs was very weak (Fig. 2a), whereas CD NPs, at an 8.4 % internal quantum yield, showed suitable PL (Fig. 2b). The CD-FeAC NPs also showed good PL data for the purposes of bio-imaging (Fig. 2c). In corresponding Raman spectra, due to the stong PL interference in the CD NPs, CD-FeAC NPs peaks showed indistinguishable D and G bands (Additional file 1: Figure S1) [43]. Transmission electron microscopy (TEM) images of FeAC, CD, and CD-FeAC NPs dispersed in aqueous solution showed successful conjugation of CD NPs with FeAC NPs, with clear contrasts (Fig. 3), compared to only carbon coated copper grid. FeAC NPs displayed the amorphous phase in the entire several-layer morphology, with distinct contrasts (Fig. 3a, b), which result is consistent with the relevant previous study [40–42] and CD NPs manifested 2–5 nm spherical and semicrystalline sizes but with some aggregated NPs with ~20 nm size (Fig. 3c, d), in the obtained atomic force microscopy (AFM) image showing a rough root mean square (RMS) of 1.865 (Additional file 1: Figure S2). As for CD-FeAC NPs, CD NPs seemed to be uniformly distributed in the FeAC sheets, and FeAC NPs had an dispersion ability of CD NPs (Fig. 3e, f), especially in light of the containment of CD NPs in the FeAC matrix [44].

For additional information relevant to in vivo mice experimentation and further clinical trials, the aqueous behaviours of FeAC, CD, and CD-FeAC NPs also are needed. Thus, their surface chemistries (e.g., zeta potentials) and hydrodynamic sizes were measured in PBS buffer and serum-free RPMI media at neutral pH (Table 2). The surface charges of FeAC NPs in the PBS buffer and serum-free RPMI media were, respectively, approximately +8 and +2.3 mV of the zeta potentials, whereas that of CD was approximately –30 mV. These results were ascribed, respectively, to the abundance of protonated amine groups (i.e., cationic clusters) and carboxylated groups. Accordingly, the zeta potentials of CD-FeAC NPs in PBS buffer and nutrient media were approximately −4.0 and −7.0 mV, respectivley. The hydrodynamic sizes of FeAC, CD, and CD-FeAC NPs averaged ~202/~337, ~10.5/~20.61, and ~362.3/~519.4 nm in PBS buffer/serum-free RPMI media at neutral pH, respectively. Generally in serum-free RPMI media, the hydrodynamic size was increased due to the strong ionic effects related to the aggregation behavior of NP colloidals. As a result, the NP’s aggregation was induced. In comparison with TEM imaging analysis, DLS data showed relatively less aggregates. It may be related to drying effect for TEM sample preparation [41, 45].

**Table 2:** Averaged zeta potential values (mV) and hydrodynamic diameters (nm) of FeAC, CD, and CD-FeAC NPs in PBS buffer and serum-free RPMI media (2.0 mg/mL) at neutral pH, as determined by dynamic light scattering (DLS)

		PBS	Serum-free RPMI media	FeAC in PBS	CD in PBS	CD-FeAC in PBS	FeAC in serum- free RPMI media	CD in serum-free RPMI media	CD-FeAC in serum- free RPMI media
Zeta potential (mV)		−4.57 ± 0.96	−9.40 ± 1.30	8.17 ± 0.62	−29.07 ± 2.03	−4.09 ± 0.46	2.29 ± 1.09	−9.18 ± 0.75	−7.22 ± 0.45
Hydrodynamic size (diameter, nm)		–	–	202 ± 59	10.5 ± 1.7	362.3 ± 365.7	337 ± 77	20.61 ± 2.59	519.4 ± 110.6
Polydispersity index (PDI)		–	–	0.39 ± 0.03	0.63 ± 0.02	0.53 ± 0.02	0.33 ± 0.11	0.59 ± 0.03	0.43 ± 0.04

### XRD patterns and FT-IR spectra of FeAC, CD, and CD-FeAC NPs

**Fig. 4:**
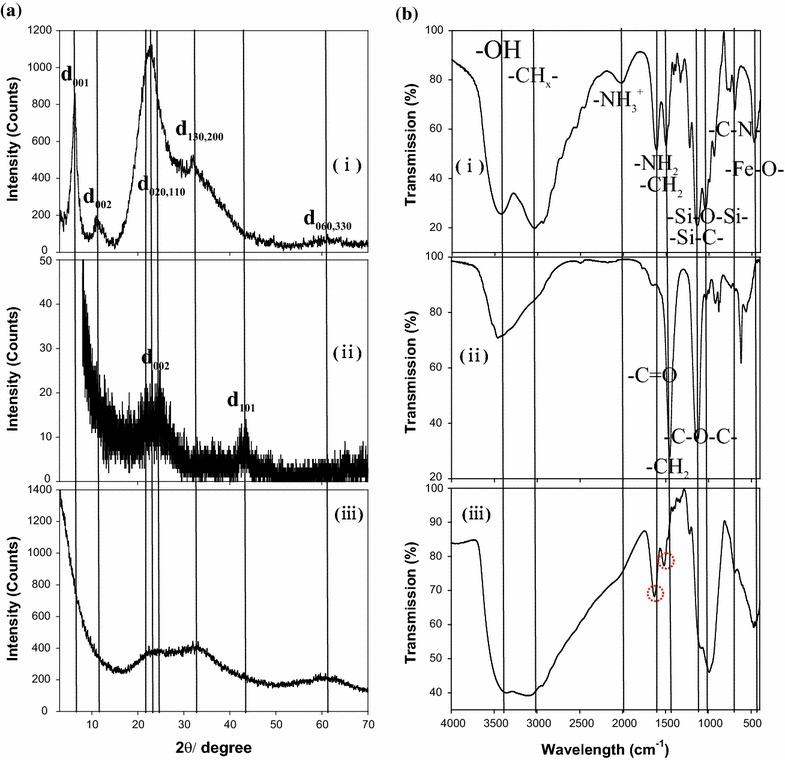
**a** Power X-ray diffraction (XRD) patterns and **b** Fourier transform infrared (FT-IR) spectra of *i* FeAC, *ii* CD, and *iii* CD-FeAC NPs in KBr-pellet mode. In **b** *iii*, the *red dotted circles* indicate amide bondings (C=O stretching, N–H deformation, and C–N stretching)

For identification and confirmation of the crystalline/amorphous phase, the power X-ray diffraction (XRD) patterns of FeAC, CD, and CD-FeAC NPs were recorded (Fig. 4a). The regular distance in the mesolamellar-structured FeAC at d_001_ was calculated to ~14.26 Å at 2θ = 6.24°, and in the broad peaks at higher angles, the distances were ~7.83, ~3.92, ~2.77, and ~1.50 Å at 2θ = 11.35, 22.69, 32.34, and 62°, respectively (Fig. 4ai), resulting in the 1:1 dioctahedral phyllosilicate [40–42]. In Fig. 4aii, the CD NPs show conventional peaks at 2θ = 24.04 and 42.87° corresponding the assignment of (002) and (101) planes of graphitic carbon. The interlayer spacing for those planes was calculated as 3.71 and 2.11 Å, respectively, indicating that the interlayer spacing of (002) was slightly shifted relative to that (3.44 Å) in bulk graphite [33]. The XRD diffraction peaks in CD-FeAC NPs matched only those in FeAC NPs (Fig. 4aiii). This can be explained by the facts that the CD NPs peaks were relatively weak and hidden.

The covalent bindings of organic-functional groups were detected by the Fourier transform infrared (FT-IR) spectra (Fig. 4b). The FT-IR peak assignments in FeAC NPs were as follows: –OH (3384 cm^−1^), –CH_x_ (3000 cm^−1^), –NH_3_^+^ (2020 cm^−1^), H_2_O (1609 cm^−1^), –CH_2_ (1487 cm^−1^), Si–C (1121 cm^−1^), Si–OH (1034 cm^−1^), Si–O–Si (1014 cm^−1^), Si–O–C (770 cm^−1^), Fe–O–Si (679 cm^−1^), and Fe–O (481 cm^−1^) (Fig. 3bi), which are in line with those reported [40–42]. The CD NPs FT-IR peak assignments were –OH (3397 cm^−1^), C=O (1599 cm^−1^), and C–O–C (1446 cm^−1^) (Fig. 4bii), indicating the abundance of oxygen-rich groups and the resultantly hydrophilic surface and partial oxidation state of the CD NPs. Significantly, in the case of CD-FeAC NPs, most of the FeAC and CD NPs peaks were recorded as overlapped. The amide-bonding characteristics in CD NPs at C=O (1620 cm^−1^) and N–H and C–N (1499 cm^−1^) were confirmed by the slight peak shift at C=O (1599 cm^−1^) [45–48].

### Cytotoxicity results of FeAC, CD, and CD-FeAC NPs

**Fig. 5:**
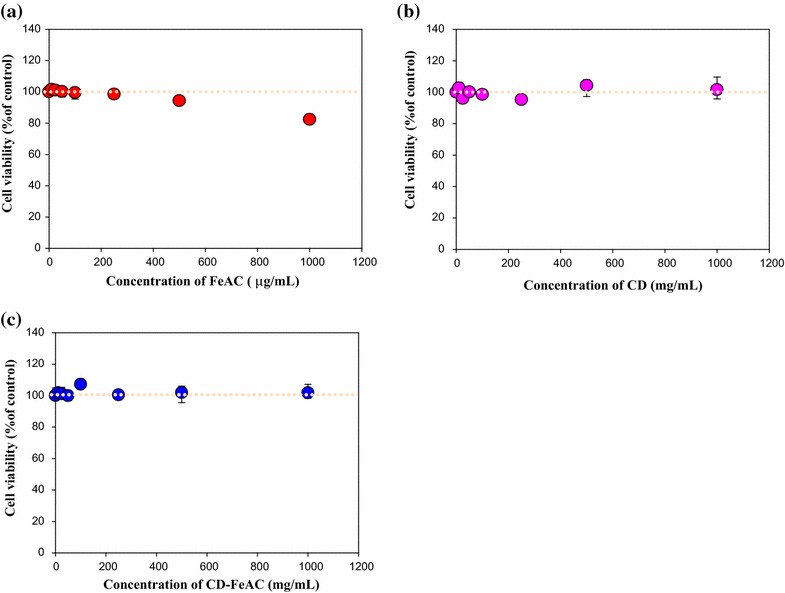
3-(4,5-dimethylthiazol-2-yl)-2,5-diphenyltetrazolium bromide (MTT) cell viability (%) of **a** FeAC, **b** CD, and **c** CD-FeAC NPs loading concentrations where *yellow dotted lines* indicate as 100 % cell viability guides

**Fig. 6:**
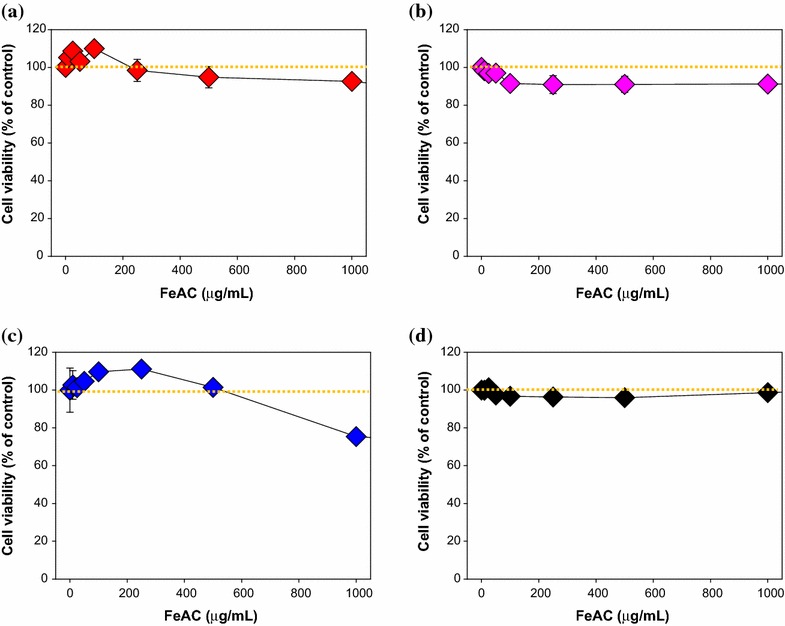
MTT cell viability assays (%) of FeAC NPs loading concentrations for **a** A549, **b** WI-38, **c** WM-266-4, and **d** CCD-986SK. Note that *yellow dotted lines* indicate as 100 % cell viability guides

**Fig. 7:**
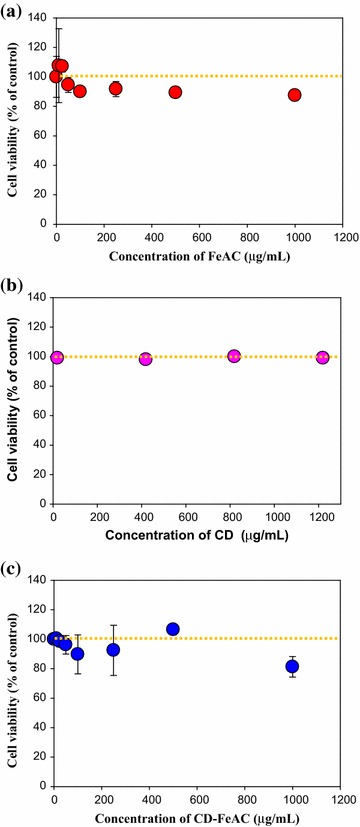
NR cell viability assays (%) of FeAC (**a**), CD (**b**), and CD-FeAC (**c**) NPs loading concentrations in HeLa cells. Note that *yellow dotted lines* indicate as 100 % cell viability guides

Based on the characterization data for FeAC, CD, and CD-FeAC NPs, MTT cytotoxic assay [29, 49] was tested according to the sample loading concentrations (Fig. 5a–c). FeAC NPs resulted in negligible cytotoxicity in normal cells but a slight (20 %) cytotoxic effect in cancer cells up to 1000 μg/mL. CD showed no cytotoxicity up to 1000 μg/mL. In CD conjugated FeAC (CD-FeAC) NPs, it was also reduced cytotoxicity due to biocompatible CD property and decreasing accessibility with Fe source in FeAC NPs. To see FeAC NPs cytotoxicity in detail, FeAC NPs were evaluated for other cancer and normal cell lines (Fig. 6), it resulted in slightly cytotoxic effects by reduced cell viability (%). Beyond mitochondria-based MTT assay, chromatin-based NR assays of FeAC, CD, and CD-FeAC according the loading concentrations in HeLa cells showed similar trends (Fig. 7).

### NPs’s observation of cellular uptake in HeLa cells

**Fig. 8:**
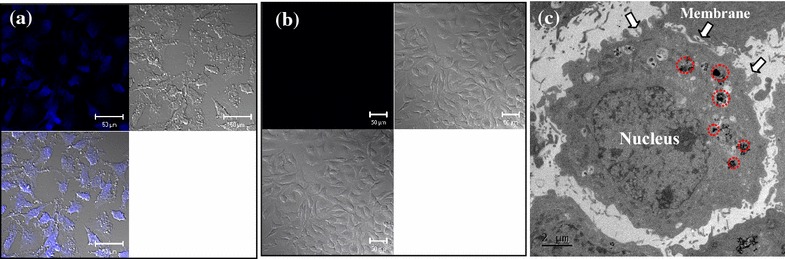
Confocal microscopy images of CD-FeAC NPs treatment in HeLa cells (**a**) and fresh HeLa cells (**b**) and where is fluorescent image in *upper-left panel*, bright field image in *upper-right panel,* and overlapped image in *bottom-left panel*, and **c** cross-sectioned transmission electron microscopy (TEM) image of FeAC NPs treatment in HeLa cells. In **b**, the *arrows* and *red dotted circles* indicate the boundary of cell membrane and the uptaken FeAC NPs, respectively, in a single HeLa cell

**Fig. 9:**
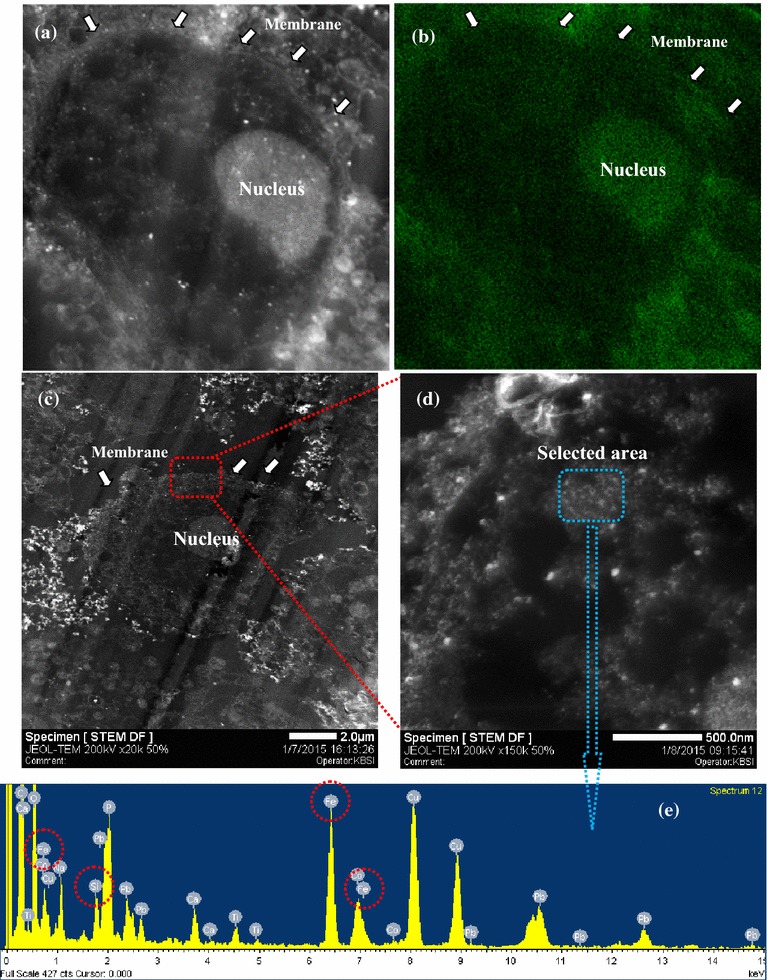
Cross-sectioned transmission electron microscopy (TEM) image (**a**) and its Fe elemental mapping with *green color* (**b**), and scanning transmission electron microscopy (STEM) image (**c**), **d** enlarged image of **c**, and its energy-dispersive X-ray (EDX) analysis (**e**) in Z-contrast mode of a single HeLa cell with treatment of FeAC NPs (200 μg/mL)

**Fig. 10:**
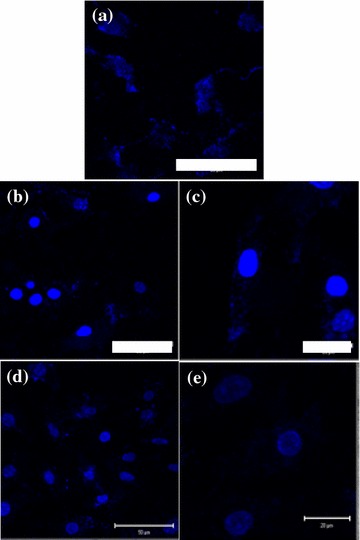
Confocal microscopic images of DAPI stained HeLa cells in the presence of FeAC NPs at 0 μg/mL (**a**), 250 μg/mL (**b**, **c**) and 500 μg/mL (**d**, **e**). *Scale bars* (**a**, **b**, **d**) 50 μm, (**c**, **e**) 20 μm

Remarkably, the cytotoxicity of CD-FeAC NPs in HeLa cells was slightly decreased at <1000 μg/mL, owing to the biocompatible CD conjugation, compared to that of FeAC treatment. Confocal microscopy images of the uptaken CD-FeAC NPs in HeLa cells and RAG cells showed a clear blue emission (Fig. 8a; Additional file 1: Figure S3a) in comparison with photos of fresh HeLa cells (Fig. 8b) and RAG cells (Additional file 1: Figure S3b). Because FeAC NPs without fluorescent materials were discerned by contrast in cross-sectioned TEM image, the intracelluar location of only FeAC clusters in cross-sectioned HeLa cells was confirmed as the cytoplasm (Fig. 8c), indicating that the uptaken FeAC NPs showed no acute cytotoxicity to the living cell’s morphology. Furthermore, it was confirmed by elemental mapping of the uptaken FeAC NPs into a single HeLa cell, showing markedly contrasted or sharp elemental peaks of Fe and Si in the presence of FeAC NPs (Fig. 9). In addition, confocal microscopy images of DAPI stained nucleus in the absence and presence of FeAC NPs were observed (Fig. 10). Only DAPI stained HeLa cells showed slightly scattered blue emission in nucleus sites (Fig. 10a) but clear blue emission in the presence of FeAC NPs showed spherical or elliptical morphology (Fig. 10b–e), interestingly, FeAC NPs may show a negligible change while nucleus staining, retaining nuclear integrity.

## Discussion

The cytotoxic results of Mg- and Ca-aminoclays were researched as non-toxic nanomaterial for bio-medical and bio-imaging [29–31]. In this study, cytotoxicity of FeAC resulted in slightly cytotoxic effects by reduced cell viability (%), due to the oxidation of H_2_O_2_ in cancer cells as a result of coordinated Fe^3+^ source to hydroxyl free radicals (∙OH) like Fenton-like reaction [50–52]. These ∙OH induced death in the cancer cell or apoptosis in vitro due to the radical-induced DNA- and cell-membrane damage. CD conjugation why it reduced cytotoxicity in FeAC NPs may be related to decreasing probability in accessibility of Fe source with cells. In detail, the reduced toxicity may be related to CD blocked the entrance or contact sites of FeAC for reactive oxygen species (ROS) generation, rather than the negatively charged surface property in CD-FeAC NPs.

In generally, anticancer agents delivered into cytosol and then, reached nucleus and directly effected in cell death [34]. So, tracking studies of CD-FeAC or FeAC NPs carriers into cells is important. As shown in Fig. 8c, FeAC NPs were existed into nucleus neighbors in HeLa cells abundantly, indicating CD-FeAC or FeAC NPs carriers can be successfully delivered target compounds into cytosol or nucleus in cells. In addition, nucleus site was negligibly damaged by FeAC NPs (Fig. 10). It is indicated that intact FeAC NPS have little cytotoxic effects for nucleus sites as well as an ability of transfection carriers [53–55]. As therapeutic agents, thus, taking into consideration the practical concentration in this study, <500 μg/mL concentration of CD-FeAC is suitable to development of transfection reagent [29, 31]. In conclusion, CD-FeAC NPs can be a useful diagnostic and therapeutic agent in providing drug-delivery-carrier with simultaneous fluorescent bio-imaging and tracking functionalities, although the use of only FeAC NPs can be given up the simultaneous bio-imaging.

## Conclusions

In summary, it has dealt that CD-FeAC NPs plays roles both in bio-imaging and as a drug-delivery carrier into human cells with little cytotoxicity as simple preparation and inexpensive sources. In close future, cinnamic acid derivatives as anticancer agent can be loaded for target cargos [56] by amide bonding between carboxylic groups in cinnamic acid derivatives and amine groups in FeAC NPs. Taking into consideration that, as noted above, the practical applied dosage of NPs is <500 μg/mL, CD-FeAC NPs is feasible for trapping of targeting drugs and proteins, because it shows cytotoxicity only to cancer cells. Conclusively, a CD-FeAC-based hybrid agent for imaging/selective anticancer platform in in vivo is currently in the planning stage.

## Experimental section

### Preparation of Fe-aminoclay (FeAC) NPs

Synthesis of FeAC NPs was carried out according to the method available in the literature [40–42]. To a 500 mL-beaker solution containing 200 mL of ethanol, 8.4 g (31.08 mmol) of FeCl_3_∙6H_2_O salt (Sigma-Aldrich, USA) was added. After complete dissolution by 10 min magnetic stirring, 13.0 mL (58.73 mmol) of 3-aminopropyltriethoxysilane (APTES, Sigma-Aldrich, USA) was added to the ferric (Fe^3+^) ethanolic solution. In the course of mixing preparatory to the sol–gel reaction, brown slurry was formed. After 6 h equilibrium in production, the FeAC product was collected by 10 min 6000×*g* centrifugation. Then, after two ethanol-washing steps, the FeAC product was oven-dried at 50 °C for 1 day. Finally, preparatory to its use, the FeAC product was ground by pestle and mortar into a brown powder.

### Preparation of carbon nanodots (CD) NPs

Organic waste solution (100 g of dried animal feces’s wastes per 10 L of three double distilled water) at 45 °C was treated by 40 kHz ultrasound for 90 min (Ultrasonics UC-05, Lab Companion, Korea) [33, 34]. It was then centrifuged at 2500 rpm for 5 min to remove large or agglomerated particles. The CD-containing supernatant was filtered twice through a 0.22 μm pore sized membrane to remove any remnant large particles, and finally dried at 45 °C.

### Conjugation of CDs with FeAC (CD-FeAC) NPs

For conjugation on the surface of FeAC NPs, CD NPs (5.0 mg) were dissolved in 1.0 mL of phosphate-buffered saline (PBS buffer, 0.01 M and pH 7.2). To induce formation of labile intermediates on the CD surfaces, 1-ethyl-3-[3-dimethylaminopropyl]carbodiimide (EDC) (2.2 mg, 11.2 μmol) and *N*-hydroxysulfo-succinimide (sulfo-NHS) (2.44 mg, 11.2 μmol) were added, and the solution was stirred in darkness for 30 min at room temperature (RT). The thus-activated CD solution (200 μL) subsequently was mixed with 1 mL of FeAC solution (10.0 mg/mL in PBS buffer) and stirred for 3 h at RT. After completion of amide-bonded conjugation, the reacted sample was washed twice with PBS buffer and incubated in 0.1 M Tris–HCl buffer (pH 7.4) for 1 h at RT with shaking (250 rpm), to cap the unreacted sulfo-NHS functionality [31, 40]. The final product (10 mg/mL) was re-suspended in PBS buffer and stored at 4 °C until use.

### Characterizations of FeAC, CD, and CD-FeAC NPs

Transmission electron microscopy (FE-TEM, Tecnai TF30 ST, FEI company, USA) images were examined. Samples had been prepared for TEM imaging by dropping a tiny pipetted amount on a carbon-coated Cu grid (300-mesh) and oven-drying at 50 °C. After 2 h bath-sonication of 2.0 mg/mL of FeAC and CD-FeAC NPs in PBS buffer and serum-free Roswell Park Memorial Institute (RPMI) media, respectively, their hydrodynamic diameter sizes and zeta potential values were measured using particle size analyzer (Zetasizer nano zs, Malvern, UK). For CD’s height and surface roughness, after 100 μL of the CD solution was placed on a silicon wafer and air-dried overnight, it was scanned by atomic force microscope (AFM, VEECO Instrument, USA). Disorder-induced D and first-order graphite G bands in samples were recorded by Raman microscopy system (NT-MDT NTEGRA Systems, USA), utilizing photoluminescence (PL) spectroscopy with a changeable UV transilluminator (DUT-260, Core Bio Systems, Korea) within the 290–410 nm excitation range. In order to check intrinsic property of quantum-like structure of CD, the quantum yield of pristine CD at 3 mg/mL was examined by spectrofluorometer (FP-8500, Jasco, Japan) following the literature [33].

The powder X-ray diffraction (XRD) patterns from 3° to 70° were obtained in 0.01 increments by micro-area X-ray diffractometry (D/MAX-2500, Rigaku, 40 kV and 300 mA) [42]. Additionally, the organic functional groups in the samples were determined according to the recorded Fourier transform infrared (FT-IR) spectra using FT-IR spectrophotometer (FT-IR 4100, Jasco, Japan) (composition: 90 wt% KBr plus 10 wt% sample in KBr-pellet mode).

### Cytotoxic evaluations of by 3-(4,5-dimethylthiazol-2-yl)-2,5-diphenyltetrazolium bromide (MTT) and neutral red (NR) assays

In order to examine cytotoxic effect of samples, two assays such as mitochondria—MTT and chromatin-based NR means were approached. The designed cell line systems were organized with cancer and normal cells (Table 1). The cytotoxicities of FeAC, CD, and CD-FeAC were measured using the EZ-Cytox Cytotoxicity assay Kit (MTT assay, Daeillab Service, South Korea) [29] and neutral red (NR) solution (Sigma-Aldrich, USA) [49]. Cells were seeded in 96-well plates to a concentration of 1 × 10^4^ cells per well with RPMI culture media. After 24 h, the RPMI culture media was replaced with various concentrations of FeAC, CD, and CD-FeAC NPs in cell free culture media prepared by two-times washing, followed by immediate 2 h bath-sonication. Cells were incubated for 24, 48, and 72 h at 37 °C, after which they were washed twice with PBS buffer. Next, 100 μL of EZ-Cytox solution (MTT assay) or NR solution (NR assay) with 25 μg/mL in serum-free RPMI media was added and incubated for 3 h. The NR solution was washed with PBS buffer before adding 100 μL of lysis solution including acetic acid, ethanol, and water (1:50:49). The absorbance spectra of formazan (MTT assay) and the released NR (NR assay) were measured with the Infinite^®^ 200 PRO (TECAN) at wavelengths of 450 and 542 nm, respectively. Cytotoxicity is expressed corresponding to MTT and NR release by untreated control cells.

### Cellular uptake observation by confocal microscopy and cross-sectioned transmission electron microscopy (TEM)

HeLa cells and RAG cells were cultured on an 8-well chamber slide with a concentration of 2 × 10^4^ cells per well and on a 6-well chamber slide with a concentration of 2 × 10^5^ cells per well for confocal microscopy (LSM510 META NLO, Carl Zeiss, Germany) and field emission transmission electron microscopy (FE-TEM, Tecnai TF30 ST, FEI company, USA), respectively. After 24 h incubation, cells were exposed to FeAC or CD-FeAC NPs for a further 24 h, and were then washed several times with PBS buffer. After drying the PBS on the 8-well chamber slide, the slide was covered with cover glass using fluorescent mounting media (DaKo). The fluorescence of CD-FeAC NPs in cells was measured under LSM510 META non-linear optic (NLO) confocal microscopy at excitation 345 nm and emission 460 nm wavelength. Cells in the 6-well plate were collected using trypsin–EDTA and washed twice with PBS buffer. Another observation of the nucleus in the FeAC NPs-treated and absence of FeAC NPs with HeLa cells, 4′,6-diamidino-2-phenylindole (DAPI, 1 μg/mL, 10 min-incubation) staining protocol was followed and observed under confocal microscopy.

As for the cross-sectioned TEM imaging, it followed the procedure available in the literature [45]. In detail, the FeAC NPs-treated HeLa cells were fixed in a 2.5 % paraformaldehyde-glutaraldehyde mixture buffered with phosphate (0.01 M and pH 7.2) for 2 h, post-fixed in 1.0 % osmium tetroxide in the same buffer for 1 h, dehydrated in graded ethanol and propylene oxide (PPO), and embedded in Epon-812. Ultra-thin sections, cut by the ULTRACUT E (Leica, Austria) ultramicrotome, were stained with uranyl acetate and lead citrate and examined under CM 20 electron microscopy (Philips, Netherlands).

## Additional file

10.1186/s12951-015-0151-z Raman spectra of FeAC, CD, and CD-FeAC NPs. **Figure S2.** Height roughness image (a) with including height (nm) analysis (b) of CD NPs by atomic force microscope (AFM). Note that in (a) the black underlining marks were employed as size guides. **Figure S3.** confocal microscopy images of CD-FeAC NPs treatment in RAG cells (a) and fresh RAG cells (b).

## Abbreviations

FeAC: Fe-aminoclay
NPs: nanoparticles
CD: carbon nanodots
MTT: 3-(4,5-dimethylthiazol-2-yl)-2,5-diphenyltetrazolium bromide
NR: neutral red
DAPI: 4′,6-diamidino-2-phenylindole
CNTs: carbon nanotubes
0D: zero-dimensional
1D: one-dimensional
2D: two-dimensional
EDS/NHS: 1-ethyl-3-(3-dimethylaminopropyl)-carbodiimide/*N*-hydroxysulfosuccinimide
PL: photoluminescent
TEM: transmission electron microscopy
AFM: atomic force microscopy
RMS: rough root mean square
RPMI: Roswell Park Memorial Institute
DLS: dynamic light scattering
XRD: X-ray diffraction
FT-IR: Fourier transform infrared
APTES: 3-aminopropyltriethoxysilane

## References

[1] Nicolas J, Mura S, Brambilla D, Mackiewicz N, Couvreur P (2013). Design, functionalization strategies and biomedical applications of targeted biodegradable/biocompatible polymer-based nanocarriers for drug delivery. Chem Soc Rev.

[2] Samal SK, Dash M, Vlierberghe SV, Kaplan DL, Chiellini E, van Blitterswijk C (2012). Cationic polymers and their therapeutic potential. Chem Soc Rev.

[3] Hubbell JA, Chilkoti A (2012). Nanomaterials for drug delivery. Science.

[4] Bansal R, Tripathi SK, Gupta KC, Kumar P (2012). Lipophilic and cationic tiphenylphosphonium grafted linear polyethylenimine polymers for efficient gene delivery to mammalian cells. J Mater Chem.

[5] Liu H, Wang H, Yang W, Cheng Y (2012). Disulfide cross-linked low generation dendrimers with high gene transfection efficacy, low cytotoxicity, and low cost. J Am Chem Soc.

[6] ChlopekJ J, Czajkowska B, Szaraniec B, Frackowiak E, Szostak K, Béguin F (2006). In vitro studies of carbon nanotubes biocompatibility. Carbon.

[7] Yu JH, Kwon S-H, Petrášek Z, Park OK, Jun SW, Shin K (2013). High-resolution three-photon biomedical imaging using doped ZnS nanocrystals. Nat Mater.

[8] Kang H, Kim S-H, Yang S-M, Park J-H (2014). Bio-inspired nanotadpoles with component-specific functionality. J Mater Chem B.

[9] Lin Y-S, Wu S-H, Hung Y, Chou Y-H, Chang C, Lin M-L (2006). Multifunctional composite nanoparticles: magnetic, luminescent, and mesoporous. Chem Mater.

[10] Park W, Yang HN, Ling D, Yim H, Kim KS, Hyeon T (2014). Multi-modal transfection agent based on monodisperse magnetic nanoparticles for stem cell gene delivery and tracking. Biomaterials.

[11] Karakoti AS, Das S, Thevuthasan S, Seal S (2011). PEGylated inorganic nanoparticles. Angew Chem-Int Edit.

[12] Ghosh D, Bagley AF, Na YJ, Birrer MJ, Bhatia SN, Belcher AM (2014). Deep, noninvasive imaging and surgical guidance of submillimeter tumors using targeted M13-stabilized single-walled carbon nanotubes. Proc Natl Acad Sci USA.

[13] Lim YY, Noh Y-W, Han JH, Cai Q-Y, Yoon K-H, Chung BH (2008). Biocompatible polymer-nanoparticle-based bimodal imaging contrast agents for the labeling and tracking of dendritic cells. Small.

[14] Wan S, Huang J, Guo M, Zhang H, Cao Y, Yan H (2007). Biocompatible superparamagnetic iron oxide nanoparticle dispersions stabilized with poly(ethylene glycol)–oligo(aspartic acid) hybrids. J Biomed Mater Res Part A.

[15] Lu X, Jiang R, Yang M, Fan Q, Hu W, Zhang L (2014). Monodispersed grafted conjugated polyelectrolyte-stabilized magnetic nanoparticles as multifunctional platform for cellular imaging and drug delivery. J Mater Chem B.

[16] Wu Y, Guo R, Wen S, Shen M, Zhu M, Wang J, Shi X (2014). Folic acid-modified laponite nanodisks for targeted anticancer drug delivery. J Mater Chem B.

[17] Thanh NTK, Green LAW (2010). Functionalisation of nanoparticles for biomedical applications. Nano Today.

[18] Avvakumova S, Colombo M, Tortora P, Prosperi D (2014). Biotechnological approaches toward nanoparticle biofunctionalization. Trends Biotechnol.

[19] Service RF (2005). Nanotechnology takes aim at cancer. Science.

[20] Kwon KC, Ryu JH, Lee J-H, Lee EJ, Kwon IC, Kim K (2014). Proteinticle/gold core/shell nanoparticles for targeted cancer therapy without nanotoxicity. Adv Mater.

[21] Burkett SL, Press A, Mann S (1997). Synthesis, characterization, and reactivity of layered inorganic-organic nanocomposites based on 2:1 trioctahedral phyllosilicates. Chem Mater.

[22] Mann S, Burkett SL, Davis SA, Fowler CE, Mendelson NH, Sims SD (1997). Sol-gel synthesis of organized matter. Chem Mater.

[23] Mann S (2009). Self-assembly and transformation of hybrid nano-objects and nanostructures under equilibrium and non-euilibrium conditions. Nat Mater.

[24] Holmström SC, Patil AJ, Butler M, Mann S (2007). Influence of polymer co-intercalation on guest release from aminopropyl-functionalized magnesium phyllosilicate mesolamellar nanocomposites. J Mater Chem.

[25] Lee Y-C, Park W-K, Yang J-W (2011). Removal of anionic metals by amino-organoclay for water treatment. J Hazard Mater.

[26] Datta KKR, Achari A, Eswaramoorthy M (2013). Aminoclay: a functional layered material with multifaceted applications. J Mater Chem A.

[27] Lee Y-C, Kim EJ, Ko DA, Yang J-W (2011). Water-soluble organo-building blocks of aminoclay as a soil-flushing agent for heavy metal contaminated soil. J Hazard Mater.

[28] Lee Y-C, Jin ES, Jung SW, Kim Y-M, Chang KS, Yang J-W (2013). Utilizing the algicidal activity of aminoclay as a practical treatment for toxic red tides. Sci Rep.

[29] Han H-K, Lee Y-C, Lee M-Y, Patil AJ, Shin H-J (2011). Magnesium and calcium organophyllosilicates: synthesis and in vitro cytotoxicity study. ACS App Mater Interfaces.

[30] Chaturbedy P, Jagadeesan D, Eswaramoorthy M (2010). pH-sensitive breathing of clay within the polyelectrolyte matrix. ACS Nano.

[31] Yang L, Lee Y-C, Kim MI, Park HG, Huh YS, Shao Y (2014). Biodistribution and clearance of aminoclay nanoparticles: implication for in vivo applicability as a tailor-made drug delivery carrier. J Mater Chem B.

[32] Yang S-T, Cao L, Luo PG, Lu F, Wang X, Wang H (2009). Carbon dots for optical imaging in vivo. J Am Chem Soc.

[33] Park SY, Lee HU, Park ES, Lee SC, Lee J-W, Jeong SW (2014). Photoluminescent green carbon nanodots from food-waste-derived sources: large-scale synthesis, properties, and biomedical applications. ACS Appl Mater Interfaces.

[34] Lee HU, Park SY, Park ES, Son B, Lee SC, Lee JW (2014). Photoluminescent carbon nanotags from harmful cyanobacteria for drug delivery and imaging in cancer cells. Sci Rep.

[35] Reuel NF, Dupont A, Thouvenin O, Lamb DC, Strano MS (2012). Three-dimensional tracking of carbon nanotubes within living cells. ACS Nano.

[36] Zhang J, Kruss S, Hilmer AJ, Shimizu S, Schmois Z, Cruz FDL (2014). A rapid, direct, quantitative, and label-free detector of cardiac biomarker troponin T using near-infrared fluorescent single-walled carbon nanotube sensors. Adv Healthc Mater.

[37] Zheng XT, He HL, Li CM (2013). Multifunctional graphene quantum dots-conjugated titanate nanoflowers for fluorescence-trackable targeted drug delivery. RSC Adv.

[38] Eda G, Lin Y-Y, Mattevi C, Yamaguchi H, Chen H-A, Chen I-S (2010). Blue photoluminescence from chemically derived graphene oxide. Adv Mater.

[39] Choi BG, Park HS, Park TJ, Yang MH, Kim JS, Jang S-Y (2010). Solution chemistry of self-assembled graphene nanohybrids for high-performance flexible biosensors. ACS Nano.

[40] Lee Y-C, Kim MI, Woo M-A, Park HG, Han J-I (2013). Effective peroxidase-like activity of a water-solubilized Fe-aminoclay for use in immunoassay. Biosens Bioelectron.

[41] Lee Y-C, Huh YS, Farooq W, Han J-I, Oh Y-K, Park J-Y (2013). Oil extraction by aminoparticle-based H_2_O_2_ activation via wet microalgae harvesting. RSC Adv.

[42] Lee Y-C, Chang S-J, Choi M-H, Jeon T-J, Ryu T, Huh YS (2013). Self-assembled graphene oxide with organo-building blocks of Fe-aminoclay for heterogeneous Fenton-like reaction at near-neutral pH: a batch experiment. Appl Catal B Environ.

[43] Zhu C, Zhai J, Dong S (2012). Bifunctional fluorescent carbon nanodots: green synthesis via soy milk and application as metal-free electrocatalysts for oxygen reduction. Chem Commun.

[44] Narayanamoorthy B, Balaji S (2015). Physicochemical characterization of amino functionalized clay/Nafion nanocomposite film with embedded platinum nanoparticles for PEM fuel cells. Appl Clay Sci.

[45] Kim S, Lee Y-C, Cho D-H, Lee HU, Huh YS, Kim G-J (2014). A simple and non-invasive method for nuclear transformation of intact-walled Chlamydomonas Reinhardtii. PLoS One.

[46] Vickery JL, Thachepan S, Patil AJ, Mann S (2009). Immobilisation and encapsulation of functional protein–inorganic constructs. Mol BioSyst.

[47] Patil AJ, Li M, Dujardin E, Mann S (2007). Novel bioinorganic nanostructures based on mesolamellar intercalation or single-molecule wrapping of DNA using organoclay building blocks. Nano Lett.

[48] Choi M-H, Hwang Y, Lee HU, Kim B, Lee G-W, Oh Y-K (2014). Aquatic ecotoxicity effect of engineered aminoclay nanoparticles. Ecotox Environ Safe.

[49] Repetto G, del Peso A, Zurita JL (2008). Neutral red uptake assay for the estimation of cell viability/cytotoxicity. Nat Protoc.

[50] López-Lázaro M (2007). Dual role of hydrogen peroxide in cancer: possible relevance to cancer chemoprevention and therapy. Cancer Lett.

[51] Jomova K, Valko M (2011). Advances in metal-induced oxidative stress and human disease. Toxicology.

[52] Xu C, Yuan Z, Kohler N, Kim J, Chung MA (2009). FePt nanoparticles as an Fe reservoir for controlled Fe release and tumor inhibition. J Am Chem Soc.

[53] Panda JJ, Varsheny A, Chauhan VS (2013). Self-assembled nanoparticles based on modified cationic dipeptides and DNA: novel systems for gene delivery. J Nanobiotechnol.

[54] Tagalakis AD, Kenny GD, Bienemann AS, McCarthy D, Munye MM, Taylor H (2014). PEGylation improves the receptor-mediated transfection efficiency of peptide-targeted, self-assembling, anionic nanocomplexes. J Control Release.

[55] Wang Y-H, Fu Y-C, Chiu H-C, Wang C-Z, Lo S-P, Ho M-L (2013). Cationic nanoparticles with quaternary ammonium-functionalized PLGA-PEG-based copolymers for potent gene transfection. J Nanopar Res.

[56] De P, Balta M, Bedos-Belval F (2011). Cinnamic acid derivatives as anticancer agents: a review. Curr Med Chem.

