# Supplementary material for: In-vitro cytotoxicity assessment of carbon-nanodot-conjugated Fe-aminoclay (CD-FeAC) and its bio-imaging applications
Source: J Nanobiotechnology. 2015 Nov 26;13:88. doi: 10.1186/s12951-015-0151-z (PMC4662025; doi:10.1186/s12951-015-0151-z)
Supplement: Supplementary file 1 — 10.1186/s12951-015-0151-z Raman spectra of FeAC, CD, and CD-FeAC NPs. Figure S2. Height roughness image (a) with including height (nm) analysis (b) of CD NPs by atomic force microscope (AFM). Note that in (a) the black underlining marks were employed as size guides. Figure S3. confocal microscopy images of CD-FeAC NPs treatment in RAG cells (a) and fresh RAG cells (b). [file 12951_2015_151_MOESM1_ESM.doc]

Supplementary Information

*In-vitro* cytotoxicity assessment of carbon-nanodot-conjugated

Fe-aminoclay (CD-FeAC) and its bio-imaging applications

Kyoung Suk Kang^1†^, Hyun Uk Lee^2†^, Moon Il Ki m^3^, So Young Park ^2^, Sung-Jin Chang ^4^, Ji- Ho Park ^5^, Yun Suk Huh^6^, Jouhahn Lee ^2^, Mino Yang^7^, Young-Chul Lee^3*^and Hyun Gyu Park^1*^

^1^Department of Chemical and Biomolecular Engineering (BK21+ program), KAIST, 291 Daehakno, Yuseong-gu, Daejeon 305-701, Republic of Korea

^2^Advanced Nano-Surface Research Group, Korea Basic Science Institute (KBSI), Daejeon 305-333, Republic of Korea

^3^Department of BioNano Technology, Gachon University, 1342 Seongnamdaero, Sujeong-gu, Seongnam-si, Gyeonggi-do 461-701, Republic of Korea

^4^Department of Chemistry, Chung-Ang University, 84 Heukseok-ro, Dongjak-gu, Seoul 156-756, Republic of Korea

^5^Department of Bio and Brain Engineering (BK21+ program), KAIST, 291 Daehakno, Yuseong-gu, Daejeon 305-701, Republic of Korea

^6^Department of Biological Engineering, College of Engineering, Inha University, Incheon 402-751, Republic of Korea

^7^Division of analytical research, Korea Basic Science Institute (KBSI), Gangneung 200-701, Republic of Korea

**^*^**Correspondence: [dreamdbs@gachon.ac.kr](mailto:dreamdbs@gachon.ac.kr) (Y.-C. Lee); [hgpark1@kaist.ac.kr](mailto:hgpark1@kaist.ac.kr) (H. G. Park)

^†^These authors contributed equally to this work

**D band**

**G band**

**Figure S1.** Raman spectra of FeAC, CD, and CD-FeAC NPs.


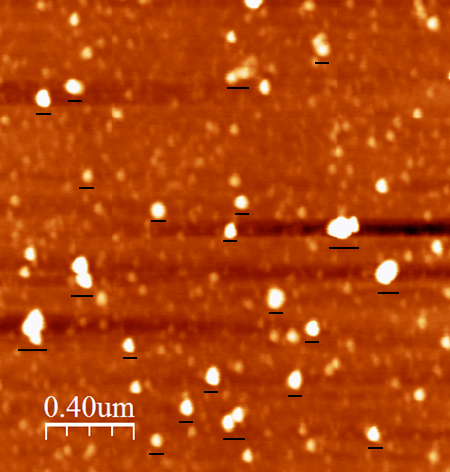

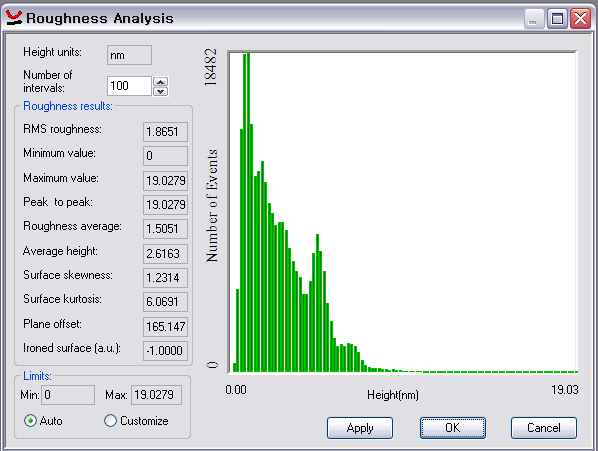


**(a)**

**(b)**

**Figure S2.** Height roughness image (a) with including height (nm) analysis (b) of CD NPs by atomic force microscope (AFM). Note that in (a) the black underlining marks were employed as size guides.

**(b)**


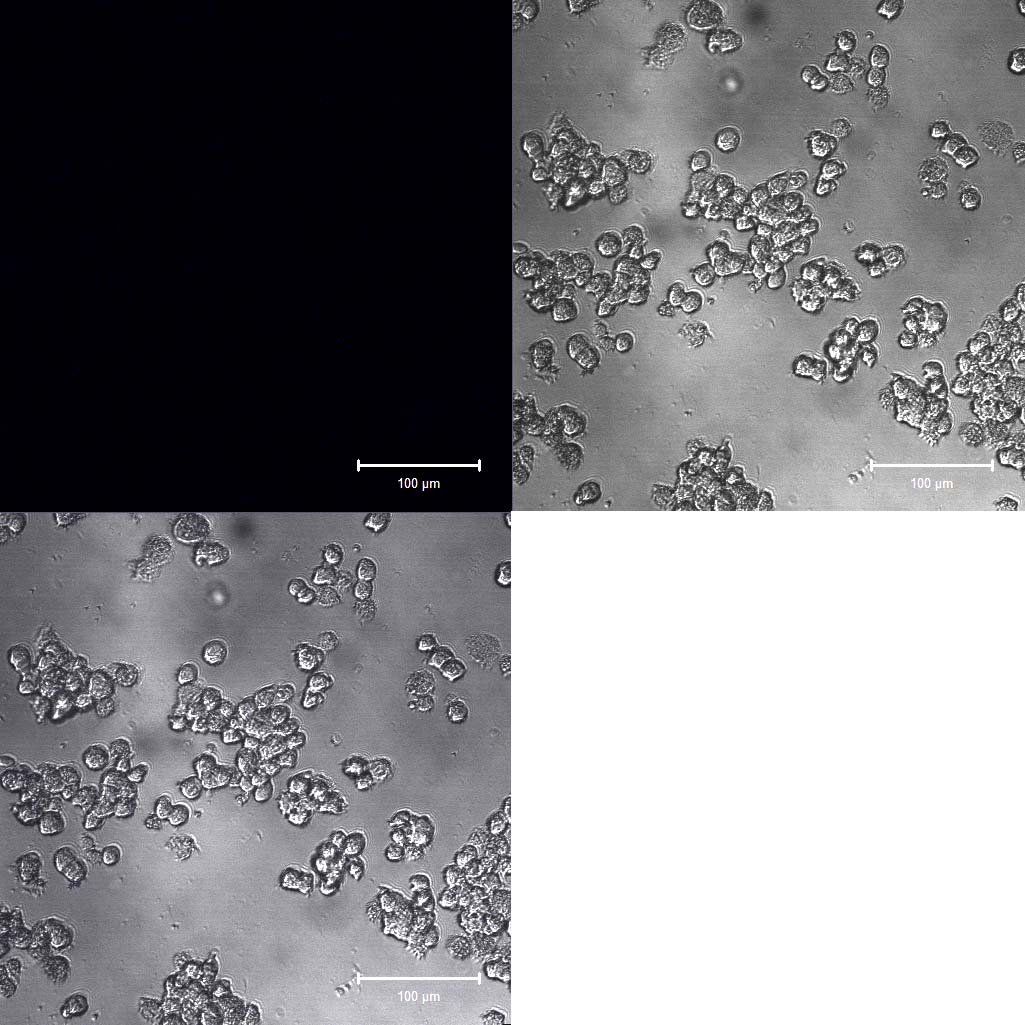


**(a)**


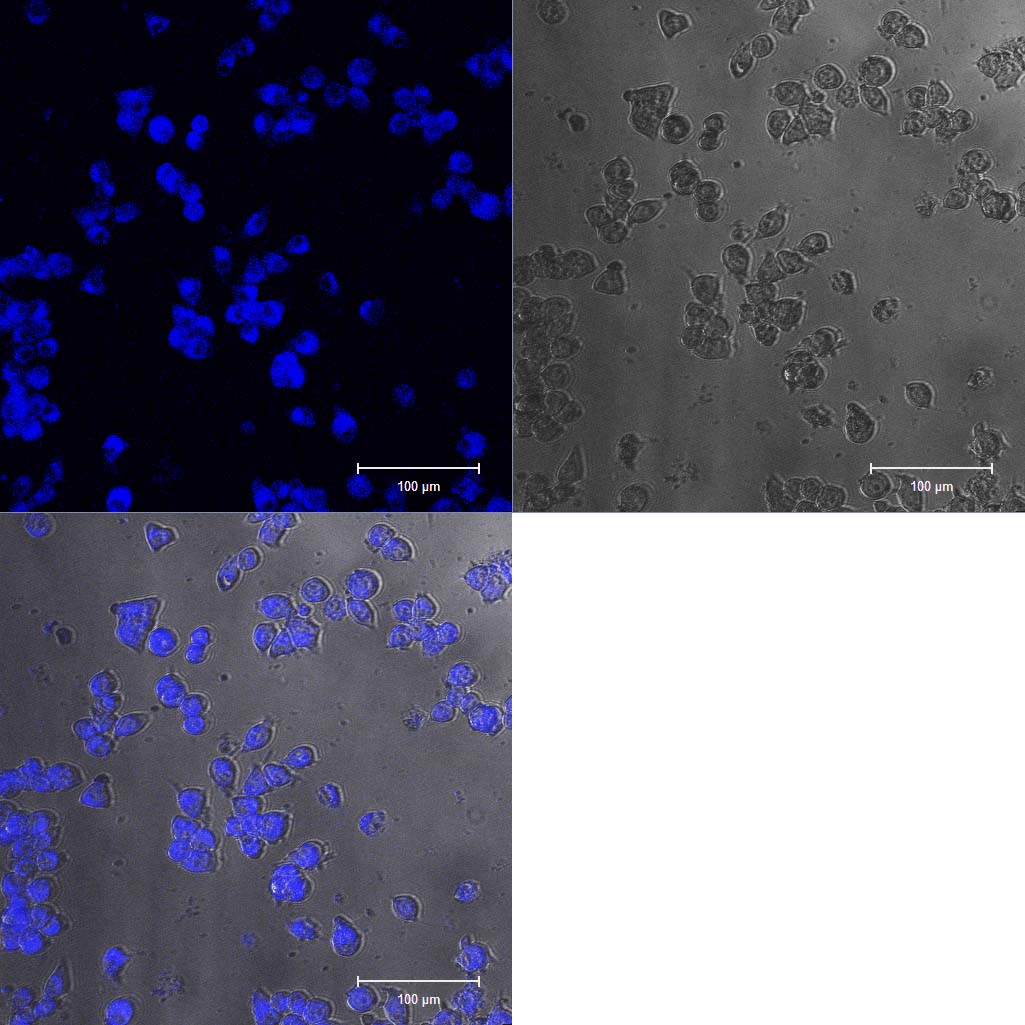


**(a)**

**(b)**

**Figure S3** confocal microscopy images of CD-FeAC NPs treatment in RAG cells (a) and fresh RAG cells (b).
